# Phase 1 study of sirolimus in combination with oral cyclophosphamide and topotecan in children and young adults with relapsed and refractory solid tumors

**DOI:** 10.18632/oncotarget.12904

**Published:** 2016-10-25

**Authors:** Kieuhoa T. Vo, Erin E. Karski, Nicole M. Nasholm, Shelly Allen, Fabienne Hollinger, W. Clay Gustafson, Janel R. Long-Boyle, Stephen Shiboski, Katherine K. Matthay, Steven G. DuBois

**Affiliations:** ^1^ Department of Pediatrics, UCSF Benioff Children's Hospital, University of California, San Francisco School of Medicine, San Francisco, CA, USA; ^2^ Department of Clinical Pharmacy, UCSF Benioff Children's Hospital, University of California, San Francisco School of Medicine, San Francisco, CA, USA; ^3^ Department of Epidemiology and Biostatistics, UCSF Benioff Children's Hospital, University of California, San Francisco School of Medicine, San Francisco, CA, USA

**Keywords:** phase 1, mTOR, sirolimus, topotecan, cyclophosphamide

## Abstract

**Purpose:**

To determine the maximum tolerated dose (MTD), toxicities, and pharmacodynamics effects of sirolimus combined with oral metronomic topotecan and cyclophosphamide in a pediatric population.

**Materials and Methods:**

Patients who were 1 to 30 years of age with relapsed/refractory solid tumors (including CNS) were eligible. Patients received daily oral sirolimus and cyclophosphamide (25-50 mg/m^2^/dose) on days 1-21 and oral topotecan (0.8 mg/m^2^/dose) on days 1-14 in 28-day cycles. Sirolimus steady-state plasma trough concentrations of 3-7.9 ng/mL and 8-12.0 ng/mL were evaluated, with dose escalation based on a 3+3 phase 1 design. Biomarkers of angiogenesis were also evaluated.

**Results:**

Twenty-one patients were treated (median age 18 years; range 9-30). Dose-limiting toxicities included myelosuppression, ALT elevation, stomatitis, and hypertriglyceridemia. The MTD was sirolimus with trough goal of 8-12.0 ng/mL; cyclophosphamide 25 mg/m^2^/dose; and topotecan 0.8 mg/m^2^/dose. No objective responses were observed. Four patients had prolonged stable disease > 4 cycles (range 4-12). Correlative biomarker analyses demonstrated reductions in thrombospondin-1 (*p*=0.043) and soluble vascular endothelial growth factor receptor-2 plasma concentrations at 21 days compared to baseline.

**Conclusions:**

The combination of oral sirolimus, topotecan, and cyclophosphamide was well tolerated and biomarker studies demonstrated modulation of angiogenic pathways with this regimen.

## INTRODUCTION

The mammalian target of rapamycin (mTOR) is a ubiquitous serine threonine kinase that is involved in the regulation of cell cycle, angiogenesis and apoptosis. [1] Sirolimus (also known as rapamycin) is an mTOR inhibitor originally approved for immunosuppression in patients who received renal transplants. [1] More recently, the mTOR pathway has been shown to be upregulated in many pediatric solid tumors. [2, 3] As such, mTOR inhibitors have been investigated in preclinical and clinical studies of childhood cancer. In murine models, single agent sirolimus has been shown to inhibit tumor growth *in vivo* in rhabdomyosarcoma, Ewing sarcoma, medulloblastoma, glioblastoma, neuroblastoma, and osteosarcoma. [4–7] Preclinical trials looking at the combination of sirolimus and cyclophosphamide have also revealed therapeutic enhancement in xenograft models of pediatric solid tumors suggesting that mTOR inhibitors have the potential to augment the activity of conventional chemotherapy drugs. [8] Indeed, a recent clinical trial in pediatric patients with relapsed rhabdomyosarcoma demonstrated benefit of the combination of temsirolimus, vinorelbine, and cyclophosphamide. [9] In addition to direct effects on tumor cells, sirolimus has also been shown to reduce tumor angiogenesis. [7, 10, 11]

Intravenous preparations of topotecan and cyclophosphamide have been shown to be active in pediatric solid tumors, particularly Ewing sarcoma, neuroblastoma, and rhabdomyosarcoma. [12–16] Historically, this chemotherapy combination has been administered in pulses at 3-4 week intervals. However, recent data suggest that relatively low-dose, continuous chemotherapy (“metronomic”) administered over prolonged periods may be effective as well. [17] This approach has been hypothesized to work by targeting endothelial cells and thus providing a form of antiangiogenic therapy. [17] Phase 1 trials of oral cyclophosphamide and topotecan have been well tolerated when given in a continuous low-dose or “metronomic” schedule, with myelosuppression being dose limiting. [18]

The current report describes the results of a pediatric phase 1 study of sirolimus administered in combination with oral topotecan and cyclophosphamide to children and young adults with refractory or recurrent solid tumors. We pursued this combination based upon preclinical data demonstrating additive activity of sirolimus in combination with chemotherapy, the antiangiogenic properties associated with both metronomic chemotherapy and mTOR inhibition, and a desire to develop a fully oral combination for patients with advanced cancer who prefer to be treated largely at home. The primary aims of the study were to describe the toxicities and to recommend a phase 2 trough concentration of sirolimus when administered on a protracted schedule in combination with oral topotecan and cyclophosphamide. Secondary endpoints included an assessment of antitumor activity and pharmacodynamic markers of antiangiogenic effect.

## RESULTS

### Patient characteristics

Characteristics of the 21 enrolled patients are shown in Table 1. All patients had measurable disease by Response Evaluation Criteria in Solid Tumors (RECIST). One patient had received previous therapy with another mTOR inhibitor, ridaforolimus. The number of prior therapy regimens for study subjects are shown in Table 2. In the heavily pretreated cohort, patients received a median of 3 prior treatment regimens (range, 2 to 13). Patients received a median of 1 cycle (range, 1 to 12) of protocol therapy (Table 2).

**Table 1:** Patient characteristics

	**All Patients** (***N*=21)**
**Median age, years (range)**	18 (9-30)
**Male:Female**	15:6
**Diagnosis**	
Osteosarcoma	7
Ewing sarcoma	3
Rhabdomyosarcoma	3
Brain Tumor	2^a^
Neuroblastoma	1
Other	5^b^
**Disease Status**	
Measurable by RECIST	21 (100%)
**Prior Therapy**	
mTOR Inhibitor	1 (5%)
Cyclophosphamide	9 (43%)
Topotecan	7 (33%)

^a^ One patient each with medulloblastoma and glioblastoma multiforme.

^b^ One patient each with alveolar soft part sarcoma, desmosplastic small round cell tumor, esthesioneuroblastoma, myxofibrosarcoma, and undifferentiated sarcoma.

**Table 2:** Summary of prior treatment regimens, dose escalation, and dose-limiting toxicities (DLT)

Subject ID	Number of Prior Regimens	Evaluable for Cycle 1 DLT?	Cycle 1 DLT?	Number of Cycles Received	Description of DLT
**Dose Level 1**: sirolimus goal 3-7.9 ng/mL; cyclophosphamide 25 mg/m^2^; topotecan 0.8 mg/m^2^					
01	11	Yes	No	1	
02	3	Yes	No	11	
03	2	Yes	No	1	
**Dose Level 2**: sirolimus goal 8-12 ng/mL; cyclophosphamide 25 mg/m^2^; topotecan 0.8 mg/m^2^					
04	2	Yes	Yes	1	Cycle 1 DLT, prolonged thrombocytopenia
05	4	No^a^	No	1	
06	6	No^b^	No		
07	4	Yes	No	12	Subsequent cycle DLT, grade 4 hypertriglyceridemia
08	3	Yes	No	1	
09	8	Yes	No	4	Subsequent cycle DLT, grade 3 stomatitis
10	3	Yes	No	6	
11	3	Yes	No	1	
**Dose Level 3**: sirolimus goal 8-12 ng/mL; cyclophosphamide 50 mg/m^2^; topotecan 0.8 mg/m^2^					
12	Unknown	Yes	No	1	
13	3	Yes	No	1	
14	2	Yes	Yes	1	Cycle 1 DLT, prolonged ALT elevation
15	3	No^c^	No	1	
16	3	No^d^	No^d^	1	
17	13	No^a^	No	1	
18	6	No^a^	No	2	
19	8	No^a^	No	1	
20	5	Yes	Yes	3	Cycle 1 DLT, prolonged neutropenia
21	2	No^c^	No	1	

^a^ Sirolimus trough level not in range within 12 days of beginning protocol therapy.

^b^ Patient withdrew consent after the first day of protocol therapy.

^c^ Patient off study due to early progression in cycle 1.

^d^ Patient missed labs to document timely count recovery, cannot exclude DLT.

### Dose escalation and toxicity

Dose escalation and observed dose-limiting toxicities (DLTs) are summarized in Table 2. Three patients were enrolled and evaluable on dose level 1, none of whom had first cycle DLT. Eight patients were subsequently enrolled on dose level 2, two of whom were not evaluable for DLT. One patient withdrew consent after the first day of protocol therapy and the second patient did not reach sirolimus goal trough by day 12 of cycle 1. Of the six patients evaluable for DLT in dose level 2, one patient developed dose-limiting prolonged thrombocytopenia during cycle 1. With 1 of 6 patients with first cycle DLT at dose level 2, dose level 3 was then evaluated. Ten patients were enrolled on dose level 3, six of whom were not evaluable for DLT. Three patients had delays in reaching target sirolimus trough levels, two patients had early disease progression during cycle 1, and one patient had grade 4 thrombocytopenia during cycle 1 and declined appropriate laboratory studies to document recovery within 2 weeks of planned start of cycle 2 (patient subsequently went off study due to disease progression). Of the remaining four patients evaluable for DLT in dose level 3, two patients had first cycle DLT (grade 2 ALT elevation that did not resolve within 14 days; grade 4 neutropenia > 7 days). These results established that dose level 3 was not tolerable and the maximum tolerated dose (MTD) was determined to be dose level 2.

Table 3 provides details of toxicity reported in more than 10% of patients of the 21 patients who received at least one dose of protocol therapy. Myelosuppression, transaminase elevation, hyperlipidemia, and electrolyte disturbance were the most commonly reported laboratory abnormalities. Mucositis and gastrointestinal symptoms were the most commonly reported symptoms. The incidence of myelosuppression did not seem to increase in subsequent cycles. Nonhematologic toxicity was uncommon in subsequent cycles of therapy, although two patients at dose level 2 had subsequent cycle DLT (grade 4 hypertriglyceridemia; grade 3 stomatitis).

**Table 3:** Hematologic and nonhematologic toxicities observed in 21 patients in cycle 1 and in subsequent cycles of therapy with sirolimus, topotecan, and cyclophosphamide.^a^

	Maximum Grade Observed per Patient During Cycle 1				Maximum Grade Observed per Patient Across All Subsequent Cycles (2-12)			
	G1	G2	G3	G4	G1	G2	G3	G4
	(N=21 patients, 21 cycles)				(N=6 patients, 32 cycles)			
**Hematologic toxicity**								
Anemia	9	3	3		1	2		
Leukopenia	4	7	4	3	3	2	1	
Lymphopenia	3	3	8	1	2	3		
Neutropenia	3	3	3	4	1	1	1	1
Thrombocytopenia	3	2	6	3	2			
**Nonhematologic toxicity**								
Abdominal pain	4				2			
ALT elevation	5	1			1			
AST elevation	3	1						
Anorexia	2	3			1			
Bruising	3							
Constipation	4				1			
Dehydraton	2	1						
Diarrhea	8	1			3			
Dry skin	3							
Fatigue	6	2			4	1		
Headache	4	3				1		
Hypercholesterolemia	5				3			
Hyperglycemia	6				1			
Hypertension	4							
Hypertriglyceridemia	4	2	2				1	1
Hypoalbuminemia	3							
Hypokalemia	8				2			
Mucositis	9	2				2	1	
Nausea	11	4			3	1		
Proteinuria	3	2			4			
Rash	2	1			1			
Vomiting	10	1			1	2		

^a^ Only toxicities possibly, probably, or definitely related to therapy with sirolimus, topotecan, and cyclophosphamide and which occurred in more than 10% of patients in cycle 1 are displayed.

Four patients did not reach goal sirolimus trough concentrations in the first 12 days of therapy. Of the 17 patients that ultimately reached sirolimus trough goal in cycle 1, the median time to first sirolimus trough goal within range was 11 days (range, 4 to 19). In cycle 1, the median sirolimus dose needed to achieve a trough concentration of 3-7.9 ng/mL was 1.05 mg/m^2^ (range, 1.03 to 1.83) and the median dose need to achieve a trough concentration of 8-12 ng/mL was 2.76 mg/m^2^ (range, 1.25 to 5.29).

### Efficacy

One patient who withdrew one day after the first dose of protocol therapy was not evaluable for antitumor activity. No objective responses were observed among the 20 remaining patients evaluable for response. Six patients (30%) had a best response of stable disease, including patients with alveolar soft part sarcoma (12 cycles), desmoplastic small round cell tumor (DRSCT, 11 cycles), osteosarcoma (6 cycles), Ewing sarcoma (4 cycles), esthesioneuroblastoma (3 cycles), and glioblastoma multiforme (2 cycles). Among the four sarcoma patients with prolonged stable disease ( > 4 cycles), all reached sirolimus trough goal in the first 12 days of cycle 1 (median 8.5 days, range 4 to 12). At the time of this report, all patients have discontinued therapy due to disease progression.

**Figure 1:**
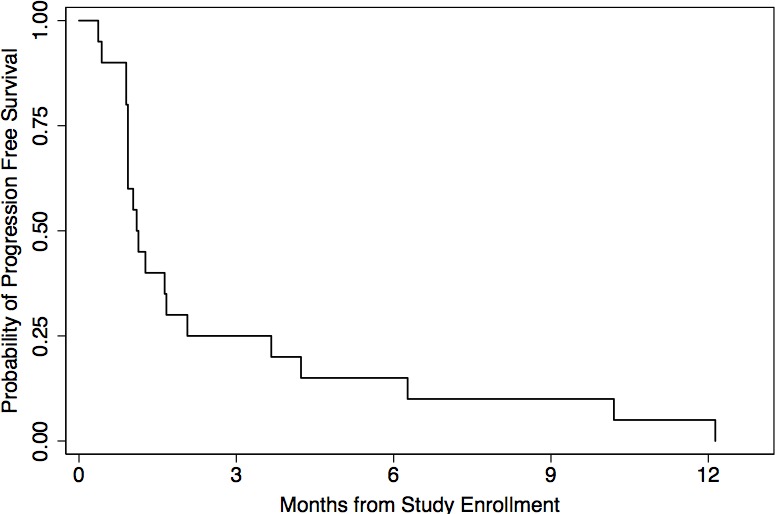
Kaplan-Meier estimated progression-free survival of 20 response-evaluable patients treated with sirolimus, topotecan, and cyclophosphamide therapy

The estimated progression-free survival (PFS) of the 20 response evaluable patients is shown in Figure 1. The median PFS was 33 days (range, 11 to 364 days). The probability of PFS at 3 and 6 months was 25% (95% CI, 9 to 45) and 15% (95% CI, 4 to 34), respectively.

### Correlative studies

**Figure 2:**
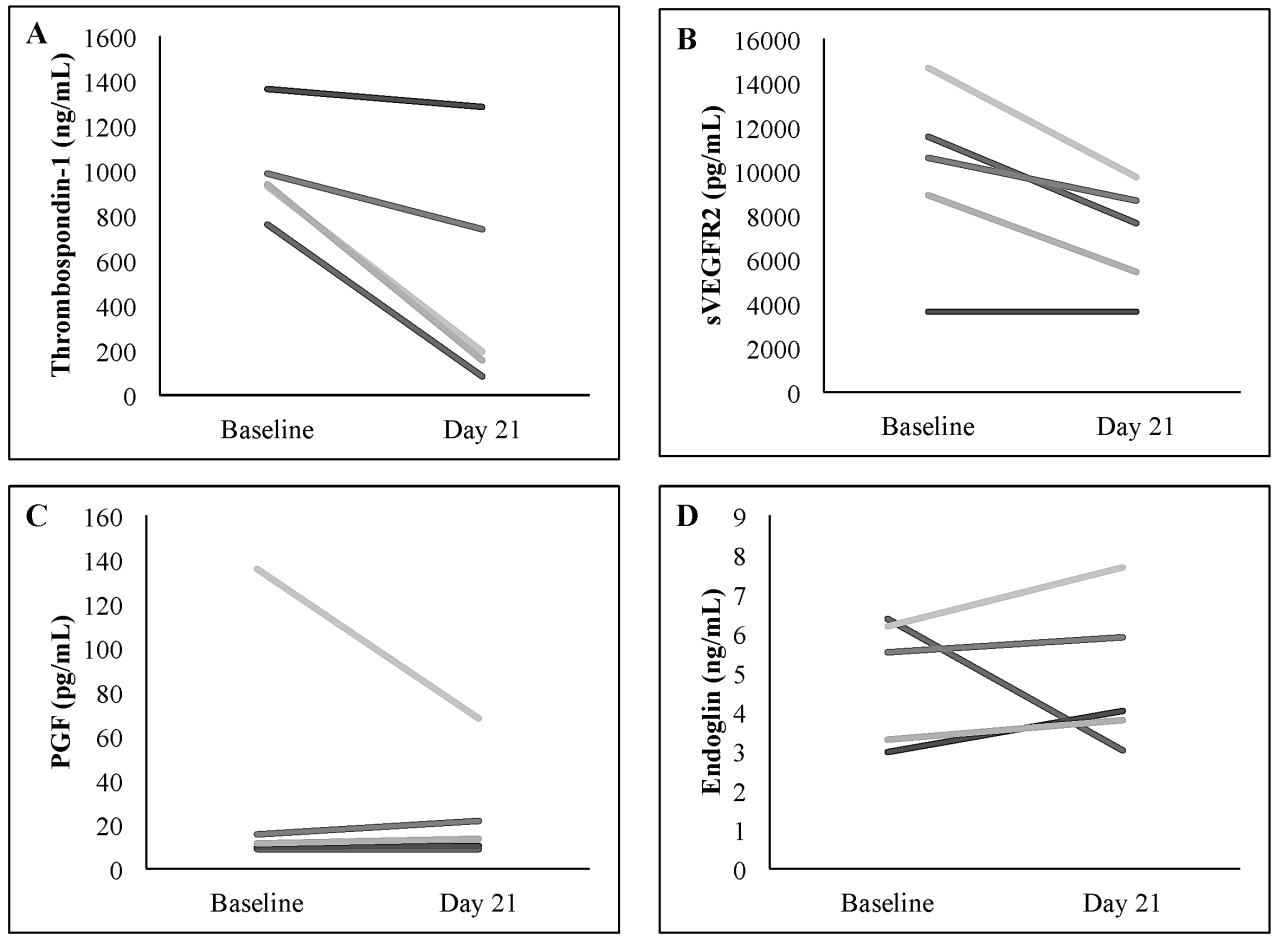
Changes in plasma **A**. thrombospondin-1, **B**. soluble VEGFR2 (sVEGFR2), **C**. placental growth factor (PGF), and **D**. endoglin concentrations from baseline to day 21 ± 2 of cycle 1 in five individual patients with paired samples.

Nineteen patients consented to the optional correlative study evaluating pharmacodynamics angiogenesis markers. Due to a range of logistical and patient issues (weekends, timing of blood draws in relation to visits to the study center, early disease progression) only thirteen samples were available at baseline and six samples were available at day 21. Over the course of cycle 1, median thrombospondin-1 decreased from 929 to 466 ng/mL, median soluble vascular endothelial growth factor receptor-2 (sVEGFR2) decreased from 11,560 to 7,569 pg/mL, median placental growth factor (PGF) decreased from 16 to 12 pg/mL, and median endoglin decreased from 5 to 4 ng/mL. Five patients had paired baseline and day 21 plasma samples available for statistical evaluation of potential changes in these markers (Figure 1). Thrombospondin-1 concentrations significantly decreased over cycle 1 (*p* = 0.043, Figure 2A). Soluble VEGFR2 concentrations trended downward over the course of cycle 1, but did not reach statistical significance (*p* = 0.057, Figure 2B). Endoglin and PGF levels did not significantly change over the course of cycle 1 (*p* = 0.50 and *p* = 0.69, Figures 2C and 2D). Given the small sample size, changes in antiangiogenesis markers were not evaluated in relation to efficacy.

## DISCUSSION

We have developed a new regimen that combines metronomic chemotherapy with an oral mTOR inhibitor. The MTD of oral combination therapy with sirolimus, topotecan, and cyclophosphamide in children and young adults with refractory and relapsed solid tumors was determined to be sirolimus on days 1-21 with steady-state trough goal concentration range of 8-12.0 ng/mL; cyclophosphamide 25 mg/m^2^/dose on days 1-21; and topotecan 0.8 mg/m^2^/dose on days 1-14. This oral 3-drug regimen was well tolerated in this heavily pretreated population. No unexpected toxicities were observed. Overall, the most common toxicity was myelosuppression, which was reversible and manageable. In general, common symptoms of mucositis and gastrointestinal events, including diarrhea, nausea, and vomiting, were low-grade. There were no objective antitumor responses in this trial. Several patients with a variety of different tumors, including four patients with sarcoma subtypes, may have benefited as evidenced by stable disease for multiple cycles, though timing of disease evaluations may have over-estimated duration of disease control. Of note, one patient with stable disease for 12 months had alveolar soft part sarcoma, a histology that may show more indolent growth, making it difficult to draw firm conclusions about the role this therapy played in stabilizing her disease.

A previous phase 1 study that evaluated metronomic oral dosing of the combination of cyclophosphamide and topotecan in pediatric patients determined the MTD to be oral cyclophosphamide (50 mg/m^2^/dose) and oral topotecan (0.8 mg/m^2^/dose) administered for 14 consecutive days in 21-28-day cycles. [18] Reversible hematologic dose-limiting toxicities (neutropenia and thrombocytopenia) were similar to those seen in this trial, [18] although the MTD of the cyclophosphamide in our trial was lower (25 mg/m^2^/dose), which may have been attributable to the addition of sirolimus; differences in length of administration (14 *vs*. 21 days on our trial); or overlap in elimination pathways between sirolimus and cyclophosphamide. While non-hematologic toxicities greater than grade 3 were not observed with metronomic cyclophosphamide and topotecan alone, the grade 3 and 4 toxicities of mucositis and hypertriglyceridemia seen in patients treated on the current study are expected toxicities of sirolimus, or other sirolimus analogues, such as temsirolimus and everolimus. [19–22] Despite the fact that the median time to reach sirolimus steady-state was 11 days, subsequent cycle DLTs were uncommon suggesting that delays in reaching steady-state in cycle 1 did not result in falsely concluding that a higher dose level was tolerable. While our study did not investigate other schedules of metronomic therapy, a schedule of oral cyclophosphamide and topotecan administered for 7 days every other week may have maximized drug exposure while decreasing hematologic toxicities.

In clinical trials, sirolimus has been combined with various cytotoxic chemotherapy agents, including intensive AML induction chemotherapy (mitoxantrone, etoposide, cytarabine); [23] gemcitabine; [24] vinblastine; [25] and paclitaxel. [26] Of particular interest, in a phase 2 trial of advanced sarcomas, patients received daily sirolimus (4 mg/dose) and oral cyclophosphamide (200 mg/dose) administered daily for 7 days every other week in 28-day cycles. Myelosuppression was a common toxicity reported with this combination. [27] Serious adverse events attributed to this therapy occurred in 11% of patients and included infection, pneumonitis, and thrombosis. [27] Patients were followed for a minimum of 6 months and 21% (10/47) of evaluable patients were progression-free for at least 6 months. [27] The 6-month PFS in patients receiving sirolimus and cyclophosphamide is higher than what was found in our study, which may be attributable to differences in the patient populations between the two clinical trials.

We examined correlative biomarkers in this trial to improve our understanding of the effect of this combination on angiogenesis pathways. While plasma thrombospondin, endoglin, PGF, and soluble VEGFR2 levels have been used as angiogenesis biomarkers in many previous studies of VEGF receptor tyrosine kinase (RTK) inhibitors, [28] little work has evaluated these markers in patients receiving mTOR inhibitors and/or metronomic chemotherapy. As in previous studies with RTK inhibitors, our combination therapy resulted in decreases in soluble VEGFR2 concentrations. [28] In addition, our study showed a significant decrease in thrombospondin-1 concentration, similar to an adult study of metronomic cyclophosphamide in advanced malignancies. [29, 30] Thrombospondin-1 is a modulator of angiogenesis and is hypothesized to play a role in mediating angiogenic activity of metronomic chemotherapy. [31] Previous studies of metronomic chemotherapy in pediatric patients showed that an elevated baseline thrombospondin-1 concentration may correlate with clinical benefit. [32, 33] Our sample size precluded a similar analysis in this study. We did not detect changes in PGF and endoglin in this study. The clinical significance of these changes in potential surrogate markers requires further evaluation.

One limitation of our study was difficulty in reaching sirolimus goal steady-state concentrations in a timely manner. Four out of 21 patients (19%) were deemed inevaluable for cycle 1 DLT assessment due to delays in reaching sirolimus goal trough concentrations by day 12 of treatment. This experience has been reported by other groups. For example, Morgenstern and colleagues reported difficulty in achieving target sirolimus trough concentration in the range of 10-15 ng/mL in pediatric patients treated with sirolimus and vinblastine. [25] In their study, sirolimus trough levels were monitored weekly during the first cycle and then at the start of subsequent cycles once in target range. Overall, only 10 of 14 patients achieved the target concentration after a mean of 3 weeks (range, 1 to 8 weeks). [25] Of all measured sirolimus concentrations, 27% were within the target range, whereas 14% were > 15 ng/mL and 59% were < 10 ng/mL. [25] This considerable inter-patient variability in dose-concentration relationship is consistent with previous reports of the variable pharmacokinetics of sirolimus. [34] Likewise in the current study, we observed a wide range of sirolimus doses needed to achieve the desired trough concentrations. These findings suggest that newer sirolimus analogues (“rapalogues”) with more consistent pharmacokinetic properties might be better suited in the context of a study targeting specific trough concentrations.

In conclusion, the combination of oral sirolimus, topotecan, and cyclophosphamide was well tolerated in this heavily pre-treated population. However, in the context of this phase 1 study, it is unclear if the addition of sirolimus improved on the anti-tumor effect of metronomic cyclophosphamide and topotecan which previously demonstrated some activity against recurrent pediatric solid tumors (partial response in one patient with neuroblastoma and a patient with medulloblastoma with prolonged stable disease). [18] The convenience associated with oral administration, evidence of modulation of angiogenesis pathways, and our findings of several patients with prolonged stable disease suggest that this regimen may be considered for patients with advanced sarcoma seeking a palliative regimen.

## MATERIALS AND METHODS

### Patients

Patients were eligible for participation if they were 12 months to 30 years of age, had histologic diagnosis of solid tumor (including brain tumors) with measurable or evaluable disease, and had no known curative options. Patients were required to have a Karnofsky (age > 16 years) or Lansky (age < 16 years) performance score of 50 or more and to have recovered from previous therapy. Patients previously treated with sirolimus, topotecan, or cyclophosphamide as single agents were eligible. Patients previously treated with two of the three drugs were eligible, though patients previously treated with all three agents in combination were excluded. Patients previously treated with sirolimus analogues (e.g. temsirolimus, everolimus, or ridaforolimus) were also eligible. Patients were required to have adequate baseline bone marrow (absolute neutrophil count > 750/μL; platelet count > 75,000/μL for patients without bone marrow involvement or platelet count > 25,000/μL for those with known bone marrow metastatic disease), renal, hepatic, pulmonary, and central nervous system function according to defined protocol criteria. Patients were required to have serum fasting triglyceride and cholesterol levels both < 300 mg/dL.

Exclusion criteria included: pregnancy or breastfeeding; concurrent use of strong CYP3A4 inducers or inhibitors; concurrent use of enzyme-inducing anticonvulsants; concurrent use of corticosteroids not on a stable or decreasing dose for 7 days prior to enrollment; uncontrolled infections; or history of allergic reaction to compounds of similar composition to sirolimus, topotecan, or cyclophosphamide.

The protocol was approved by the University of California, San Francisco Committee on Human Research. Written informed consent (and assent when applicable) was obtained for all patients. This trial was registered with ClinicalTrials.gov number NCT01670175.

### Treatment and evaluations

The dose escalation schema is summarized in Table 2. Patients received daily oral sirolimus and cyclophosphamide on days 1-21 in a 28-day cycle. This was combined with oral topotecan given once daily on days 1-14. Sirolimus dosing was based on steady-state plasma trough concentrations with a starting goal level of 3-7.9 ng/mL in dose level 1 and goals levels of 8-12.0 ng/mL in subsequent dose levels. The topotecan dose was 0.8 mg/m^2^/dose in all dose levels. The cyclophosphamide dose was 25 mg/m^2^/dose for dose levels 1 and 2 and 50 mg/m^2^/dose in dose level 3. Calculations for body surface area were capped at 2 m^2^ for cyclophosphamide and topotecan. Initial sirolimus loading and maintenance doses were based on the patient's weight, age, and assigned dose level (Table 4). Of note, sirolimus is available commercially as an oral solution (1 mg/mL) and in tablet form (1 mg and 2 mg tablets). Both formulations were available for study subjects and were chosen at the discretion of the treating physician. Sirolimus trough levels were drawn 3-4 days following a loading dose. If the level was in the desired range, a repeat level was drawn 3-4 days following the initial level. Once a stable level within the goal range was obtained two times in a row, levels were obtained once per cycle unless a change in concomitant medication or liver function had the potential to impact sirolimus metabolism. Adjustments to sirolimus maintenance and loading doses were based on calculations provided in the package insert. [35] Patients without disease progression or unacceptable toxicity could receive up to two years of therapy.

**Table 4:** Sirolimus loading and maintenance dosing by assigned dose level, age, and weight

	Targeted Sirolimus Trough 3-7.9 ng/mL	Targeted Sirolimus Trough 8-12.0 ng/mL
**Age < 13 years *and* Weight < 40 kg**	∙ Initial loading dose: 3 mg/m^2^ on day 1 ∙ Initial maintenance dose: 1 mg/m^2^ once daily starting on day 2 of each cycle	∙ Initial loading dose: 3 mg/m^2^ on day 1 ∙ Initial maintenance dose: 1 mg/m^2^ divided twice daily starting on day 2 of each cycle
**Age > 13 years *or* Weight > 40 kg**	∙ Initial loading dose: 3 mg on day 1 ∙ Initial maintenance dose: 1 mg once daily starting on day 2 of each cycle	∙ Initial loading dose: 6 mg on day 1 ∙ Initial maintenance dose: 2 mg once daily starting on day 2 of each cycle

Patients had routine physical examinations and surveillance laboratory testing to evaluate for toxicity. Toxicities were graded according to the National Cancer Institute (NCI) Common Terminology Criteria, version 4.0. DLT was defined as any of the following that were attributed as at least possibly related to study therapy: grade 4 neutropenia with fever or grade 4 neutropenia for > 7 days; platelet transfusion for a platelet count < 10 × 10^9^/L or for clinical bleeding on 2 separate days within a 7 day period; myelosuppression that delayed start of subsequent cycle by > 14 days; any grade 3 or 4 non-hematological toxicity with the specific exclusion of grade 3 nausea and vomiting < 3 days, grade 3 diarrhea < 3 days, grade 3 mucositis or stomatitis < 3 days, grade 3 alanine/aspartate aminotransferase (ALT/AST) that returned to grade < 1 or baseline prior to the start of the next treatment cycle, grade 3 fever, grade 3 infection, grade 3 electrolyte abnormalities responsive to oral supplementation within 7 days, grade 3 or 4 hypertriglyceridemia that returned to grade < 2 within 7 days of drug interruption, grade 3 or 4 hypercholesterolemia responsive to lipid lowering medication within 35 days, and grade 3 hyperglycemia that returned to < grade 2 or baseline prior to start of next treatment cycle.

Patients underwent disease evaluation at baseline and then at the end of cycles 1 and 3, and then every third cycle. For patients with measurable disease, tumor response was evaluated by RECIST. [36] For patients with neuroblastoma, a modification of the International Neuroblastoma Response Criteria was used that utilized meta-iodobenzylguanidine (MIBG) response by Curie score and RECIST for soft tissue disease. [37, 38]

### Biomarker studies

Plasma was obtained at baseline and day 21 ± 2 of cycle 1 in consenting patients to assess the impact of study therapy on biomarkers of angiogenesis. Thrombospondin-1, endoglin, PGF, and sVEGFR2 levels were measured by ELISA using commercially available kits (R&D Systems, Minneapolis, MN).

### Dose escalation strategy and statistical methods

The primary endpoint for determining dose escalation was DLT attributed to study therapy in cycle 1. A standard 3 + 3 phase 1 dose escalation design was utilized, with escalation starting at dose level 1. Patients were considered evaluable for dose escalation decisions if: they (1) received > 17 of the 21 of the planned doses of sirolimus and cyclophosphamide AND (2) received > 11 of the 14 of the planned doses of topotecan AND were followed until they met toxicity criteria to proceed to cycle 2. In addition, patients who experienced DLT at any time after the first doses of medication were considered evaluable. Lastly, patients must have had at least one sirolimus trough level in goal range within the first 12 days of cycle 1 to be considered evaluable for dose escalation/de-escalation purposes at that dose level.

Changes in angiogenesis biomarkers obtained at baseline and at the end of cycle 1 were assessed using Wilcoxon signed rank test for all paired data available at baseline and day 21 ± 2. PFS was defined as the time from enrollment to first occurrence of disease progression or death. Patients without an event were censored at the time of last patient contact. We estimated PFS with the Kaplan-Meier method and reported the median PFS and PFS at 3 and 6 months for the treatment group. All statistical analyses were performed using Stata, version 13 (StataCorp, College Station, TX).
